# Disentangling the comorbidity between allergic disease and type 1 diabetes using genetically informative designs

**DOI:** 10.1016/j.jacig.2025.100519

**Published:** 2025-06-23

**Authors:** Awad I. Smew, Tong Gong, Ralf Kuja-Halkola, Cecilia Lundholm, Arvid Harder, Yi Lu, Lars Sävendahl, Paul Lichtenstein, Bronwyn K. Brew, Catarina Almqvist

**Affiliations:** a Department of Medical Epidemiology and Biostatistics, Karolinska Institutet, Stockholm, Sweden; b Department of Women’s and Children’s Health, Karolinska Institutet, Stockholm, Sweden; c Pediatric Endocrinology Unit at Astrid Lindgren Children’s Hospital, Karolinska University Hospital, Stockholm, Sweden; d School of Medicine and Public Health, University of Newcastle, Newcastle, Australia; e Pediatric Allergy and Pulmonology Unit at Astrid Lindgren Children’s Hospital, Karolinska University Hospital, Stockholm, Sweden

**Keywords:** Allergic rhinitis, asthma, eczema, epidemiology, type 1 diabetes, genetics

## Abstract

**Background:**

Previous research has demonstrated co-occurrence of asthma and type 1 diabetes in children, but the relationship is not as clear between allergic rhinitis or eczema and type 1 diabetes. Shared familial factors could explain a comorbidity, but the genetic overlap remains to be examined.

**Objective:**

The aim was to further the etiologic understanding of the comorbidity between allergic disease and type 1 diabetes.

**Methods:**

A Swedish population-based cohort of 3 million children born 1987-2017 was linked to nationwide registers. Associations between each allergic disease and type 1 diabetes were estimated within individuals and the familial coaggregation between relatives. For the genetic overlap, linkage disequilibrium score regression was applied on the basis of genome-wide association studies. In genotyped individuals from the Swedish Twin Registry, polygenic risk scores were developed to test the prediction of genetic risk of one disease on the phenotype of the other.

**Results:**

Asthma, allergic rhinitis, and eczema were associated with type 1 diabetes (odds ratio [95% confidence interval], 1.11 [1.07-1.15] for asthma, 1.23 [1.19-1.27] for allergic rhinitis, and 1.31 [1.26-1.35] for eczema). Familial coaggregation was only detected for asthma or allergic rhinitis, not for eczema. Linkage disequilibrium score regression and polygenic risk score analysis yielded little evidence for a genetic overlap.

**Conclusions:**

Allergic diseases and type 1 diabetes seem to co-occur in individuals. For asthma and allergic rhinitis, this association existed also between relatives indicating a shared etiology but was not evident for eczema. No strong signals of a genetic overlap using molecular genetic approaches were uncovered.

Asthma and other related allergic diseases, including allergic rhinitis and eczema, are among the most frequent chronic illnesses in children and are in general characterized by a T_H_2-mediated inflammation.^1^ In contrast, type 1 diabetes is a less common, lifelong childhood-onset autoimmune condition due to a T_H_1-mediated immune response.^2^ T_H_1 and T_H_2 immunity have previously been proposed to be mutually inhibitory systems.^3^ However, there is epidemiologic evidence for the oversimplification of this dichotomy, with an increasing number of cohort studies, meta-analyses, and reviews demonstrating the co-occurrence of asthma and type 1 diabetes.^4^, ^5^, ^6^, ^7^, ^8^, ^9^ In contrast, fewer and smaller studies have been conducted on the relationship between allergic rhinitis or eczema and type 1 diabetes, and results are conflicting, with positive,^10^^,^^11^ inverse,^12^, ^13^, ^14^, ^15^ or no^16^, ^17^, ^18^ associations found.

Although a causal relationship may be an explanation for the co-occurrence of allergic disease and type 1 diabetes, with the presence or treatment of one disease increasing the risk of the other, as has been elsewhere suggested,^5^^,^^19^ an alternative hypothesis is that the comorbidity shares etiologic factors. For instance, the increasing incidence of the diseases over the past decades^20^^,^^21^ has been attributed to changes in environmental determinants, related to microbial exposure to allergens and infections, diet, pregnancy, or birth.^22^ This is in line with observations demonstrating that asthma and type 1 diabetes seem to cluster in countries,^23^ perhaps owing to common risk factors. In a previous study, we demonstrated comorbidity of asthma and type 1 diabetes within the same individual (co-occurrence) and among siblings and cousins (familial coaggregation), thereby providing evidence for a shared liability.^9^ Conversely, no coaggregation of allergic rhinitis or eczema and type 1 diabetes was found among twins^24^ and has not yet been investigated across other types of relatives.

Shared genetic factors could exist between allergic disease and type 1 diabetes given their high heritability; the aggregation of the respective diseases within families has been confirmed,^25^^,^^26^ and genome-wide association studies (GWAS) have identified associated genetic variants.^27^, ^28^, ^29^, ^30^, ^31^ The allergic diseases share a number of these with each other,^32^ whereas type 1 diabetes also has a common genetic background with several other autoimmune diseases, according to GWAS and immune cell expression profiling.^33^ Nevertheless, although the genetic liability has been established separately for the diseases, the proportion of genetic or environmental factors explaining the comorbidity, as well as the genetic overlap between them, remains unclear.

To disentangle the comorbidity between allergic disease and type 1 diabetes, our aims were, first, to assess the co-occurrence and familial coaggregation between each allergic disease (asthma, allergic rhinitis, eczema) and type 1 diabetes; second, to decompose the association into genetic and environmental contributions via quantitative genetic modeling; and third, to understand the potential shared genetic architecture of the diseases using molecular approaches that are based on GWAS summary statistics and individual-level genotype data. Consistency between findings across complementary methods could help to triangulate evidence^34^ and further elucidate knowledge gaps.

## Methods

### Study design

#### Population-based cohort

**Fig 1:**
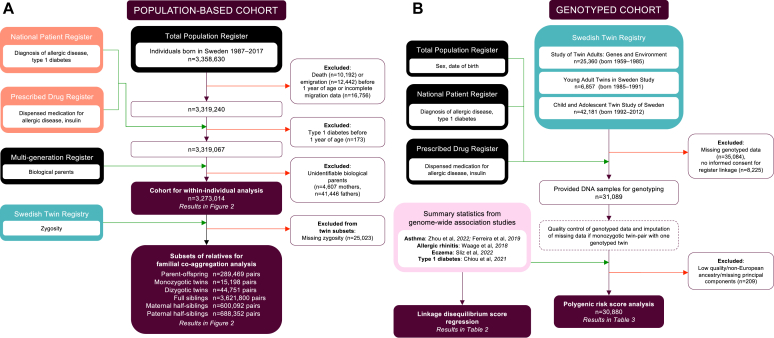
**A,** Flowchart of population-based cohort of individuals born in Sweden, 1987-2017. **B,** Flowchart of molecular genetic parts of study based on genotyped cohort of individuals from Swedish Twin Registry and inclusion of summary statistics from GWAS.

We constructed a population-based cohort by linking pseudonymized data from multiple national sources using unique personal identification numbers (Fig 1, *A*).^35^ All children registered as born in Sweden between January 1, 1987, and December 31, 2017 (n = 3,358,630), were followed through the year 2021. Individuals were identified from the Total Population Register held by Statistics Sweden, which provided information on date of birth, sex, migration, death, and family relations. We excluded individuals with invalid migration data; who died, emigrated, or developed (to avoid inclusion of cases of neonatal diabetes) type 1 diabetes before 1 year of age; or who had unidentifiable biological parents. Within this analytic cohort (n = 3,273,014), we identified subsets of relatives including all pairs of parent–offspring (any, mother, father), twins, full siblings, and maternal or paternal half-siblings. Twins in the cohort were linked to the Swedish Twin Registry (STR) for information on zygosity, either based on genotyping or questionnaires on similarity.^36^ All individuals were further linked to sources from the National Board of Health and Welfare including the National Patient Register for data on inpatient (from 1987 on) and outpatient (from 2001 on) hospital visits; and the Prescribed Drug Register for information (from July 2005 on) on dispensed medication prescriptions. Table E1 in the Online Repository available at www.jaci-global.org provides more information on national registers.

#### Genotyped cohort

The aforementioned national register information was also available through 2016 for individuals included in previously established twin studies in the STR,^37^ including the Study of Twin Adults: Genes and Environment (STAGE, n = 25,360), the Young Adult Twins in Sweden Study (YATSS, n = 6,857), and the Child and Adolescent Twin Study of Sweden (CATSS, n = 42,181). Individuals included in these 3 studies who had provided DNA samples for genotyping of single-nucleotide variants at enrollment formed the basis of our genotyped cohort (n = 30,880, Fig 1, *B*), which was used for construction and application of polygenic risk scores (PRS; more detailed information is available in the Methods section in the Online Repository available at www.jaci-global.org).

### Ethics statement

The Swedish Ethical Review Authority granted the ethical approval for this study. Informed consent was obtained from all individuals (or their parents if the study participant was under 15 years of age) in the genotyped cohort from the STR and was waived by the Swedish Ethical Review Authority for the population-based cohort because of the register-based nature of the study with inclusion of a large proportion of the entire Swedish population.

### Disease definitions

Cases of asthma, allergic rhinitis, and eczema were defined using validated algorithms^38^^,^^39^ of diagnoses and medication combinations from the National Patient Register and Prescribed Drug Register (see Table E2 in the Online Repository available at www.jaci-global.org). In a *post hoc* exploratory analysis, we categorized asthma into allergic asthma if either allergic rhinitis or eczema also were present, and nonallergic asthma if no concomitant allergic disease was registered. Type 1 diabetes was defined as any diagnosis of type 1 diabetes or any dispensation of insulin prescription, both before 18 years of age to restrict the cohort to childhood-onset disease.^40^

### PRS

In brief, PRS are weighted counts of genetic risk alleles for a specific trait in an individual derived from results of publicly available GWAS (discovery sets), then applied in independent genotyped cohorts (target sets). We calculated PRS, which is based on the SBayesR method,^41^ for each allergic disease and type 1 diabetes using the original summary statistics from the largest, most recent GWAS of asthma (3 separate scores: any, childhood onset, adult onset),^27^^,^^28^ allergic rhinitis,^29^ eczema,^30^ and type 1 diabetes,^31^ respectively, provided from each publication. More detailed information regarding the GWAS and predictive ability of each PRS on the respective trait is available in the Methods section in the Online Repository available at www.jaci-global.org (see Table E3 and Fig E1, also in the Online Repository).

### Statistical analysis

#### Within-individual and familial coaggregation analyses

Logistic regression was used to estimate the odds ratio (OR) for the association between each allergic disease (independent variable) and type 1 diabetes (dependent variable). Crude and sex- and birth year–adjusted results are presented as ORs with 95% confidence intervals (CIs). For the familial coaggregation, we estimated the OR of type 1 diabetes in relatives of individuals with each allergic disease, repeated in each subset of relatives. Models were additionally adjusted for the relative’s own allergic diseases status, thereby taking into account the possibility of direct effects of the allergic disease on type 1 diabetes within the relative. Positive associations indicate that shared familial factors may contribute to the co-occurrence of the two diseases. Results that are adjusted for direct effects add further support for a shared familial liability.^42^ Given differences in genetic and environmental sharing (see Table E4 in the Online Repository available at www.jaci-global.org), differences in estimates between relatives can shed light on which type of familial factors may contribute to the association. Data on all possible relative pairs were used; that is, each individual was included both as having a relative and as being the relative. Using family identification numbers, sandwich estimators for standard errors were applied to account for clustering within families.

Two sensitivity analyses of within-individual associations were conducted. First, we reran analyses in a restricted cohort born 1987 to 2011, allowing for a minimum of 10 years’ follow-up for all. Second, to see if individuals with incomplete follow-up were biasing the results, we excluded children who died or emigrated before the end of follow-up. Finally, we performed a sensitivity analysis for parent–offspring familial coaggregation analyses; the subset of parent–offspring pairs was broadened to identify any Swedish-born parent to an offspring in the cohort, irrespective of the parental date of birth (ie, including parents born before 1987) to increase statistical power.

#### Disease correlations within and between relatives

Quantitative genetic modeling can estimate the contribution of genetic or environmental influences on a studied trait. To determine if this type of modeling was feasible, we calculated tetrachoric correlations within and between diseases and individuals under a liability threshold framework. These included phenotypic correlations (between disease, within individual), intraclass correlations (within disease, between individuals) for each allergic disease and type 1 diabetes separately, and cross-relative, cross-trait correlations (between disease, between individuals). We also calculated concordance rates between relatives—that is, the proportion of individuals with the disease if the relative also had the same disease (univariate) or the other disease (bivariate). On the basis of these results, we determined that it was not reasonable to decompose familial sharing into latent genetic and environmental factors and therefore refrained from performing that analysis. All aforementioned analyses in the population-based cohort were performed by Stata v17.0 software (StataCorp).

#### Linkage disequilibrium score regression

Using the same GWAS summary statistics of allergic disease and type 1 diabetes as in the PRS calculation, we performed linkage disequilibrium (LD) score regression.^43^^,^^44^ This allowed us to estimate the single nucleotide polymorphism (SNP)-based heritability as well as the genetic correlation between each allergic disease and type 1 diabetes. A genetic correlation different from 1 indicates a positive or negative genetic overlap between the diseases. Analyses were performed bu LD score regression command lines in Python v2.7.5.

### PRS

ORs and 95% CIs were estimated by logistic regression with robust variance estimators to account for the correlated nature of the twin data, and they represent the risk of one disease (eg, type 1 diabetes) associated with a 1 standard deviation (SD) increase in PRS of the other disease (eg, asthma). We modeled the PRS for each allergic disease as a predictor of type 1 diabetes and the PRS for type 1 diabetes as a predictor of each allergic disease. Models are presented crude and adjusted for sex and birth year, as well as dealing with potential effect modification by target set and population stratification by including interactions terms for each twin study (STAGE, YATSS, CATSS) and the top 5 principal components, thus allowing each twin study to have unique adjustments for these principal components. Analyses were performed by SAS v9.4 (SAS Institute).

## Results

In our population-based cohort of 3 million children (Fig 1, *A*), the prevalence of asthma was 14.9% with mean age at onset 8.5 years (SD 7.5), allergic rhinitis 28.9% (11.8 years, SD 8), eczema 16.2% (8.7 years, SD 8.1), and type 1 diabetes 0.6% (9.3 years, SD 4.4). Similar distributions of disease were present across all subsets of relatives (Table I).

**Table I:** Study characteristics of population-based cohort born 1987-2017 and in each subset of relatives

Characteristic	Whole population	Parents	Monozygotic twins	Dizygotic twins	Full siblings	Maternal half-siblings	Paternal half-siblings
Total no. of unique individuals	3,199,242	192,801	14,914	43,198	2,388,977	380,009	405,934
No. of pairs in analyses	—	284,069	14,914	43,198	3,493,663	582,452	658,810
Sex							
Male	1,643,620 (51.4)	104,861 (36.9)	7,964 (53.4)	21,748 (50.3)	1,800,848 (51.6)	298,790 (51.3)	336,067 (51.0)
Female	1,555,617 (48.6)	179,208 (63.1)	6,950 (46.6)	21,450 (49.7)	1,692,815 (48.5)	283,661 (48.7)	322,740 (49.0)
Birth year							
1987-1991	564,158 (17.6)	237,970 (83.8)	2,380 (16.0)	5,012 (11.6)	508,458 (14.6)	88,593 (15.2)	105,646 (16.0)
1992-1996	534,780 (16.7)	44,217 (15.6)	3,486 (23.4)	8.508 (19.7)	632,043 (18.1)	102,271 (17.6)	115,017 (17.5)
1997-2001	429,885 (13.4)	1,882 (0.7)	2,796 (18.8)	8,454 (19.6)	537,467 (15.4)	106,732 (18.3)	109,332 (16.6)
2002-2006	484,649 (15.2)	NA	2,806 (18.8)	8,044 (18.6)	603,896 (17.3)	112,584 (19.3)	118,066 (17.9)
2007-2011	529,524 (16.6)	NA	2,776 (18.6)	6,934 (16.1)	633,424 (18.1)	93,120 (16.0)	106,561 (16.2)
2012-2017	656,246 (20.5)	NA	670 (4.5)	6,246 (14.5)	578,380 (16.6)	79,152 (13.6)	104,188 (15.8)
Asthma	475,422 (14.9)	41,360 (14.6)	2,322 (15.6)	6,985 (16.2)	503,355 (14.4)	97,747 (16.8)	108,056 (16.4)
Age (years) at onset, mean (SD)	8.5 (7.5)	18.0 (7.4)	8.4 (7.6)	7.6 (6.9)	8.3 (7.3)	8.2 (7.4)	8.4 (7.4)
Allergic rhinitis	924,023 (28.9)	115,096 (40.5)	4,092 (27.4)	11,798 (27.3)	978,702 (28.0)	183,324 (31.5)	209,681 (31.8)
Age (years) onset, mean (SD)	11.8 (8.0)	20.9 (5.2)	13.0 (7.7)	11.6 (7.5)	11.7 (7.8)	12.1 (7.8)	12.0 (7.9)
Eczema	516,993 (16.2)	40,030 (14.1)	1,991 (13.4)	6,343 (14.7)	558,320 (16.0)	92,621 (15.9)	109,924 (16.7)
Age (years) at onset, mean (SD)	8.7 (8.1)	20.2 (5.6)	10.5 (8.0)	9.1 (7.8)	8.6 (7.9)	8.8 (7.8)	8.7 (7.9)
Type 1 diabetes	20,135 (0.6)	1,831 (0.6)	79 (0.5)	258 (0.6)	22,493 (0.6)	3,938 (0.7)	4,342 (0.7)
Age (years) at onset, mean (SD)	9.3 (4.4)	10.5 (4.3)	9.8 (4.1)	9.5 (4.5)	9.4 (4.4)	9.7 (4.5)	9.6 (4.4)
Comorbid asthma and type 1 diabetes	3,259 (0.1)	282 (0.10)	13 (0.1)	40 (0.1)	3,513 (0.1)	724 (0.1)	808 (0.1)
Comorbid allergic rhinitis and type 1 diabetes	6,960 (0.2)	797 (0.28)	26 (0.2)	81 (0.2)	7,444 (0.2)	1,474 (0.3)	1,642 (0.3)
Comorbid eczema and type 1 diabetes	3,963 (0.1)	273 (0.10)	10 (0.1)	57 (0.1)	4,436 (0.1)	778 (0.1)	853 (0.1)

*NA,* Not applicable.

### Within-individual and familial coaggregation analyses

Individuals with asthma, allergic rhinitis, or eczema were at an increased risk of co-occurring type 1 diabetes (adjusted OR [95% CI], 1.11 [1.07-1.15], 1.23 [1.19-1.27], and 1.31 [1.26-1.35] respectively).

**Fig 2:**
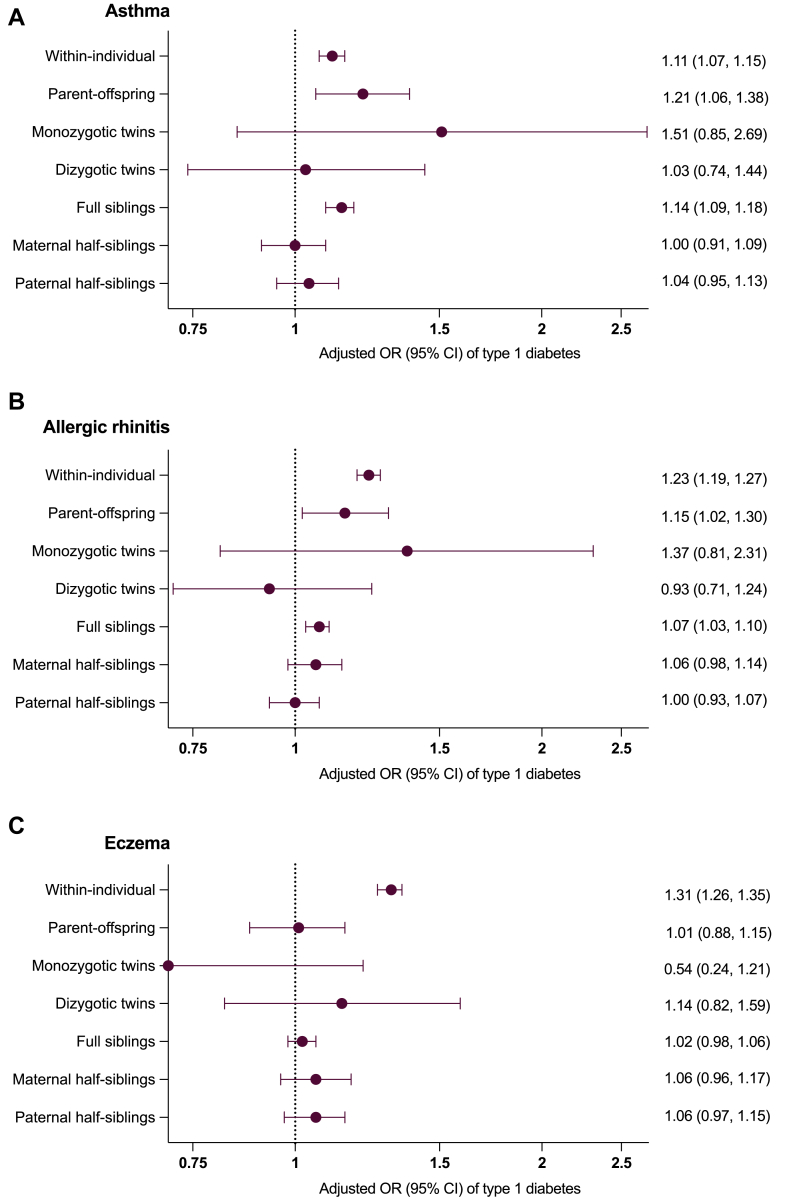
Within-individual and familial coaggregation associations between allergic diseases and type 1 diabetes. **A,** Asthma. **B,** Allergic rhinitis. **C,** Eczema. ORs are presented with 95% CIs and are adjusted for sex and birth year.

Asthma coaggregated with type 1 diabetes in parent–offspring (adjusted OR [95% CI], 1.21 [1.06, 1.38]) and full sibling pairs (1.14 [1.09, 1.18]), but not in half-siblings (Fig 2, *A*). After additional adjustment for the direct effects of asthma on type 1 diabetes, all ORs remained positive (see Table E5 in the Online Repository available at www.jaci-global.org). For allergic rhinitis (Fig 2, *B,* and see Table E6 in the Online Repository), a similar pattern of familial coaggregation was found with type 1 diabetes in parents (1.15 [1.02, 1.30]) and full siblings (1.07 [1,03, 1.10]). Familial coaggregation was not present for eczema (1.01 [0.88, 1.15] in parent–offspring, 1.02 [0.98, 1.06] in full siblings, Fig 2, *C,* and see Table E7 in the Online Repository). For twins, the statistical certainty of the estimates was low with wide CIs throughout.

Sensitivity analyses allowing for a minimum of 10 years’ follow-up or excluding children who died or emigrated yielded similar point estimates in within-individual analyses (see Table E8 in the Online Repository available at www.jaci-global.org). When including parent–offspring pairs irrespective of the parental birth year (see Table E9 in the Online Repository), results of familial coaggregation were of a similar magnitude across all allergic diseases (see Table E10 in the Online Repository).

In a *post hoc* exploratory analysis (see Table E11 in the Online Repository available at www.jaci-global.org), we found that individuals with both asthma and concomitant allergic rhinitis or eczema had an increased risk of type 1 diabetes (adjusted OR [95% CI], 1.14 [1.09, 1.18]), whereas those with asthma, but without any other allergic diseases, did not (1.03 [0.96, 1.10]).

### Disease correlations

The phenotypic correlation between asthma and type 1 diabetes was low (0.02 [0.01, 0.03]) and was similar for allergic rhinitis (0.06 [0.05, 0.06]) or eczema (0.05 [0.04, 0.06]) and type 1 diabetes. Point estimates for the intraclass correlations of each disease were in general higher among relatives of closer genetic relatedness (see Table E12 in the Online Repository available at www.jaci-global.org). However, cross-relative correlations between the allergic diseases and type 1 diabetes were essentially null (see Table E13 in the Online Repository).

### LD score regression

In the analyses that is based on GWAS summary data, SNP-based heritability was highest for childhood-onset asthma (29.8% of the variation explained). However, no genetic correlations between any of the allergic diseases and type 1 diabetes were found (*P* values ranging from .15 to .83; Table II).

**Table II:** Genetic relationship between allergic diseases and type 1 diabetes using LD score regression based on summary statistics from GWAS

Disease	GWAS sample size	Disease prevalence, sample:population (%)	No. of SNPs in analysis	h^2^-SNP∗ (SE)	Genetic correlation with type 1 diabetes (SE)	*P* value
Asthma						
Asthma	1,800,785	8.5:8.0	976,221	0.08 (0.01)	0.05 (0.03)	.15
Childhood-onset asthma	314,633	8.1:8.0	1,175,113	0.30 (0.03)	−0.05 (0.05)	.28
Adult-onset asthma	327,253	4.4:5.0	1,175,040	0.13 (0.01)	0.02 (0.04)	.64
Allergic rhinitis	38,838	27.2:28.0	1,185,127	0.12 (0.02)	0.02 (0.08)	.83
Eczema	796,661	2.8:3.0	1,188,232	0.08 (0.02)	−0.01 (0.02)	.72
Type 1 diabetes	520,580	3.64:0.5	1,213,408	0.10 (0.01)	—	—

*SE,* Standard error.

∗ SNP-based heritability was converted to liability scale with population-based prevalence from literature and sample prevalence data from summary statistics.

### PRS

In the cohort of genotyped individuals (Fig 1, *B,* n = 30,880), PRS for asthma seemed to predict type 1 diabetes (adjusted OR [95% CI] 1.34 [0.95, 1.88] any asthma, 1.34 [1.00, 1.78] childhood asthma, 1.33 [0.90, 1.96] adult asthma), although CIs were wide and included the null. In contrast, neither PRS for allergic rhinitis (1.04 [0.67, 1.62]) nor eczema (0.91 [0.67, 1.23]) predicted type 1 diabetes, and PRS for type 1 diabetes was not associated with asthma (1.00 [0.96, 1.03]) or any other allergic disease (Table III).

**Table III:** Associations between PRS and phenotypes for allergic diseases and type 1 diabetes in genotyped cohort of 30,880 individuals

Predicted phenotype	Disease of PRS	Crude OR (95% CI)	Adjusted OR (95% CI)∗
Type 1 diabetes (n = 28)	Asthma		
	Asthma	1.34 (0.96, 1.86)	1.34 (0.95, 1.88)
	Childhood-onset asthma	1.33 (1.00, 1.77)	1.34 (1.00, 1.78)
	Adult-onset asthma	1.32 (0.90, 1.93)	1.33 (0.90, 1.96)
	Allergic rhinitis	1.04 (0.68, 1.59)	1.04 (0.67, 1.62)
	Eczema	0.90 (0.66, 1.21)	0.91 (0.67, 1.23)
Asthma (n = 4,665)	Type 1 diabetes	1.00 (0.97, 1.04)	1.00 (0.96, 1.03)
Allergic rhinitis (n = 7,355)	Type 1 diabetes	1.00 (0.97, 1.03)	1.00 (0.97, 1.03)
Eczema (n = 3,655)	Type 1 diabetes	1.03 (0.99, 1.07)	1.03 (0.99, 1.07)

∗ Adjusted for sex and birth year, and including interaction terms for top 5 principal components and from which twin study in Swedish Twin Registry each individual originated.

## Discussion

In this nationwide Swedish cohort study of more than 3 million children, we applied several epidemiologic, genetically informative designs. We demonstrated co-occurrence of allergic disease and type 1 diabetes in individuals, as well as familial coaggregation of asthma or allergic rhinitis and type 1 diabetes in parents and full siblings, suggesting a shared liability. Familial coaggregation was not found for eczema and type 1 diabetes. Molecular genetic approaches yielded little evidence for a genetic overlap.

Our findings of a co-occurrence between asthma or other allergic diseases and type 1 diabetes are in line with previous literature on the subject arguing for a more nuanced stance on the T_H_1/T_H_2 balance than the previously postulated strict mutual exclusivity of the two immune responses. For instance, it has been shown that children with both asthma and type 1 diabetes have a unique pattern of cytokines conducive with a combined T_H_1/T_H_2 profile,^45^^,^^46^ that children with allergic disease have increased IL-2 levels (an important T_H_1 signaler),^47^ and that other regulatory subtypes of T cells, including T_H_17, partake in inflammation in both asthma and type 1 diabetes.^3^ Because asthma often in itself co-occurs with other allergic diseases, as was the case in our sample, it is reasonable to believe that they all be similarly related to type 1 diabetes. As indicated in our *post hoc* analysis, allergic asthma with concomitant allergic disease may be driving the overall association found between asthma and type 1 diabetes. The inverse relationships previously proposed in retrospective case–control studies of eczema and type 1 diabetes^12^, ^13^, ^14^, ^15^ could be biased as a result of underreporting symptoms of allergy in individuals with such a serious disease as type 1 diabetes. Taken together, this supports the biological plausibility of a comorbidity between allergic disease and type 1 diabetes.

We used familial coaggregation analyses to confirm an increased risk of type 1 diabetes among parents or siblings of those who have asthma.^9^^,^^48^ This strengthens evidence for a shared familial liability to the diseases, which may be either genetic or environmental, given stronger estimates among parent–offspring or full sibling pairs than half-siblings. To our knowledge, we are the first to report the presence of familial coaggregation for allergic rhinitis and type 1 diabetes, which also points to shared factors partially underlying this comorbidity. In contrast, the lack of coaggregation found for eczema and type 1 diabetes between relatives instead implies individual-level factors not shared within families explaining their co-occurrence.

After finding evidence for familial coaggregation, we aimed to understand how much of the etiology was due to genetic or environmental factors. However, the correlations found within and between twins and siblings were quite low. This, alongside the fact that quantitative genetic modeling requires larger samples the rarer the disease, hindered us from decomposing the phenotypic correlation into constituent sources. Instead, we applied two different molecular genetic methods and found little evidence for a genetic overlap.

First, using LD score regression, we found no correlation between SNPs related to any allergic disease and SNPs associated with type 1 diabetes. This finding adds to the heterogenous and inconclusive body of previous research efforts comparing specific genes of allergic diseases and type 1 diabetes. Some susceptibility loci (eg, 6q15, 12q13.2) known to be associated with asthma were also associated with type 1 diabetes;^49^ conversely, a large proportion of other loci were not.^50^ Other results suggest that common variants of genes related to the T_H_1/T_H_2 immune response were inversely associated between asthma and autoimmune disease, whereas associations for genes related to regulatory T-cell pathways had the same direction for both disease groups.^51^ This does not necessarily contradict our findings of no strong signals of a shared genetic background because LD score regression does not account for the entire genetic overlap (only SNP-based heritability), and smaller correlations may exist that did not reach statistical significance. In summary, there is little evidence for a large genetic overlap using the methods we applied, although we cannot rule out an unidentifiable weak or local genetic overlap, given a small number of specific common SNPs that may exist.

Second, using a PRS-based approach, we found a potential association, albeit with limited statistical certainty, for asthma–PRS predicting type 1 diabetes but not for type 1 diabetes–PRS predicting asthma or any other allergic disease. Similar estimates were found in a study^52^ that were based on an older GWAS for asthma^53^ (OR [95% CI] for asthma–PRS predicting type 1 diabetes 1.91 [1.58, 2.30]). If a large genetic overlap between the allergic diseases and type 1 diabetes did exist, we would, however, have expected the PRS associations to be bidirectional. Instead, our finding points to a potential causal link between asthma and type 1 diabetes where the asthma phenotype influenced by the genetic risk for asthma may in turn be associated with the development of type 1 diabetes. Genetic risk for type 1 diabetes, however, does not seem to be associated with asthma. This hypothesis is in line with a recent two-sample Mendelian randomization study that reported evidence of a causal effect of asthma on type 1 diabetes, but not for type 1 diabetes on asthma.^5^ However, we cannot rule out alternative explanations such as pleiotropic effects or differences depending on subgroups of phenotypes. Future research will therefore be instrumental in determining which factors underpin a causal relationship between asthma and type 1 diabetes. Whether the increased risk of type 1 diabetes is due to exposure to corticosteroids, including inhaler asthma medication, as has been put forward elsewhere,^19^ disease severity, or other mediating or confounding factors remains to be examined.

Thus, the observed associations between allergic diseases and type 1 diabetes in this study do not to a large extent seem to be explained by shared genetics. Given the demonstrated familial coaggregation, a shared liability, if not genetic, could perhaps be of environmental origin. Several environmental exposures may be related to both allergic disease and type 1 diabetes, such as respiratory infections in infancy, including respiratory syncytial virus, rhinovirus, or coronavirus disease 2019, gut microbiome alterations, obesity, diet, and exposure to animals.^6^ Investigation of such factors lies beyond our scope here, but we believe that ensuing research on the topic will be instrumental and may also consider other related and potentially relevant factors such as sibship size, day care attendance, or receipt of corticosteroid therapy. A nongenetic shared origin is also reasonable to assume, given the increasing incidence of both allergic disease and type 1 diabetes over the past decades, which cannot solely be explained by genetic changes, although gene–environment interactions may also be at play.

Strengths of this study include its cohort design, which is based on unambiguous linkage of multiple national registers with high-quality, real-world prospective data on the entire Swedish population, including 20,000 cases of type 1 diabetes and using validated measures of allergic disease; the identification of relatives, which enabled application of family-design methodology; and the utilization of published GWAS summary statistics and individual-level genotyped data from twin studies, allowing for converging evidence from multiple analyses.

Certain limitations must be acknowledged, however. We lacked more detailed phenotypic information on the allergic diseases such as sensitization, severity, control, or age at first symptoms, which would have been valuable, given the increasing importance placed on endotypes. Our exploratory analysis did, however, find that individuals with asthma as well as another allergic disease more often had type 1 diabetes, whereas those with asthma and no other allergic diseases did not. These findings require replication in purposively designed studies, ideally with biochemical markers of disease and assessing the impact of and effect on type 1 diabetes glycemic control.

Moreover, on the one hand, surveillance bias may have arisen if individuals with type 1 diabetes were more easily diagnosed with allergic disease because of their health care contact. On the other hand, although those with the mildest allergic disease not requiring any medical attention may be missed, our validated algorithms used for case definitions have high sensitivity. They capture allergic diseases even among those who only have medication dispensations—and, importantly, regardless of whether they have type 1 diabetes. Type 1 diabetes misclassification ought to be negligible given the need for hospitalization when it manifests.

For genetic analyses, power was insufficient for assessing coaggregation among twins or half-siblings and for performing quantitative genetic analyses. Much like other studies,^54^^,^^55^ PRS for allergic diseases had limited predictive ability for the respective allergic disease phenotypes. This could be due to issues with the underlying GWAS not accurately identifying all relevant SNPs for the phenotype of interest, modest SNP effect sizes, and phenotype heterogeneity. However, we were able to complement these findings with LD-score regression methods to estimate genetic correlation, as has been previously recommended.^56^ Both genetic analyses will be interesting to replicate when larger GWAS are available and using other phenotypic measures of T_H_2 inflammation, such as IgE levels. Finally, generalizability of results both from observational and genotyped data to populations of other ethnic distributions may be limited, especially given exclusion of non-European ancestry samples; future studies should mitigate this through replication in other or more diverse populations.

To conclude, we show that allergic diseases and type 1 diabetes co-occur in children and to a certain extent coaggregate within families, indicating a shared liability that may be environmental, given little evidence for a genetic overlap in molecular analyses. Future research efforts should focus on further understanding potential underlying mechanisms that explain the comorbidity, such as risk factors common for allergic diseases and type 1 diabetes, causal relationships, and potential biases. Regardless of the limited direct clinical application of these findings, given the small magnitude of associations, awareness of the presented comorbidity is pertinent to the management of children affected by the diseases and provides insights for continued research efforts, especially regarding disease control and treatment strategies.

**Key messages**

• In this population-based cohort study, individuals with allergic disease more often had type 1 diabetes; asthma or allergic rhinitis and type 1 diabetes coaggregated in families, indicating a shared familial liability; and the genetic overlap between allergic disease and type 1 diabetes is minimal.
• These findings expand on previous knowledge of the comorbidity. They highlight the importance of investigating shared environmental factors for future research and increase the awareness of comorbid diseases, which may guide clinical management.

## Disclosure statement

Supported by the 10.13039/501100004359Swedish Research Council (grants 2018-02640 and 2023-02327), the Strategic Research Program in Epidemiology at 10.13039/501100004047Karolinska Institutet, the Swedish Heart–Lung Foundation (grants 2018-0512 and 2021-0416), the 10.13039/501100010234Swedish Asthma and Allergy Association Research Fund (grant 2020-0008), the Foundation “Frimurare Barnhuset Stockholm,” and “H.K.H. Kronprinsessan Lovisas förening för barnasjukvård.” A.I.S. was supported through funding from the Clinical Scientist Training Programme and Medical Research Internship, both at 10.13039/501100004047Karolinska Institutet.

Data availability: Original data are held by Swedish National Board of Health and Welfare and Statistics Sweden. Because of Sweden’s data storage laws, we cannot make the data publicly available. However, any researcher can access the data by obtaining ethical approval from the Swedish Ethical Review Authority and thereafter asking the registers for the original data. Pseudonymized data can also be provided on request after providing a reasonable proposal and if an appropriate data-sharing agreement with Karolinska Institutet can be established.

Disclosure of potential conflict of interest: The authors declare that they have no relevant conflicts of interest.

## References

[1] Papi A., Brightling C., Pedersen S.E., Reddel H.K. (2018). Asthma. Lancet.

[2] Atkinson M.A., Eisenbarth G.S., Michels A.W. (2014). Type 1 diabetes. Lancet.

[3] Rabin R.L., Levinson A.I. (2008). The nexus between atopic disease and autoimmunity: a review of the epidemiological and mechanistic literature. Clin Exp Immunol.

[4] Zeng R., Wang Z., Zhang J., Liang Z., Xu C., Wang J. (2022). Type 1 diabetes and asthma: a systematic review and meta-analysis of observational studies. Endocrine.

[5] Xie J., Chen G., Liang T., Li A., Liu W., Wang Y. (2022). Childhood asthma and type 1 diabetes mellitus: a meta-analysis and bidirectional Mendelian randomization study. Pediatr Allergy Immunol.

[6] Sgrazzutti L., Sansone F., Attanasi M., Di Pillo S., Chiarelli F. (2021). Coaggregation of asthma and type 1 diabetes in children: a narrative review. Int J Mol Sci.

[7] Liljendahl M.S., Sevelsted A., Chawes B.L., Stokholm J., Bønnelykke K., Andersen Z.J. (2022). Childhood asthma is associated with development of type 1 diabetes and inflammatory bowel diseases: a Danish nationwide registry study. Sci Rep.

[8] Metsälä J., Lundqvist A., Virta L.J., Kaila M., Gissler M., Virtanen S.M. (2018). The association between asthma and type 1 diabetes: a paediatric case–cohort study in Finland, years 1981-2009. Int J Epidemiol.

[9] Smew A.I., Lundholm C., Sävendahl L., Lichtenstein P., Almqvist C. (2020). Familial coaggregation of asthma and type 1 diabetes in children. JAMA Netw Open.

[10] Lin C.H., Wei C.C., Lin C.L., Lin W.C., Kao C.H. (2016). Childhood type 1 diabetes may increase the risk of atopic dermatitis. Br J Dermatol.

[11] Fsadni P., Fsadni C., Fava S., Montefort S. (2012). Correlation of worldwide incidence of type 1 diabetes (DiaMond) with prevalence of asthma and atopic eczema (ISAAC). Clin Respir J.

[12] Cardwell C.R., Shields M.D., Carson D.J., Patterson C.C. (2003). A meta-analysis of the association between childhood type 1 diabetes and atopic disease. Diabetes Care.

[13] Stene L.C., Rønningen K.S., Bjørnvold M., Undlien D.E., Joner G. (2010). An inverse association between history of childhood eczema and subsequent risk of type 1 diabetes that is not likely to be explained by HLA-DQ, PTPN22, or CTLA4 polymorphisms. Pediatr Diabetes.

[14] Schmitt J., Schwarz K., Baurecht H., Hotze M., Fölster-Holst R., Rodríguez E. (2016). Atopic dermatitis is associated with an increased risk for rheumatoid arthritis and inflammatory bowel disease, and a decreased risk for type 1 diabetes. J Allergy Clin Immunol.

[15] Narla S., Silverberg J.I. (2019). Association between atopic dermatitis and autoimmune disorders in US adults and children: a cross-sectional study. J Am Acad Dermatol.

[16] Duran C., Ediger D., Ersoy C., Coskun N.F., Selimoglu H., Ercan I. (2008). Frequency of atopy and allergic disorders among adults with type 1 diabetes mellitus in the southern Marmara region of Turkey. J Endocrinol Invest.

[17] Karavanaki K., Tsoka E., Karayianni C., Petrou V., Pippidou E., Brisimitzi M. (2008). Prevalence of allergic symptoms among children with diabetes mellitus type 1 of different socioeconomic status. Pediatr Diabetes.

[18] Jasser-Nitsche H., Varga E.M., Borkenstein H.M., Höntzsch J., Suppan E., Weinhandl G. (2017). Type 1 diabetes in children and adolescents is not associated with a reduced prevalence of atopy and allergic diseases. Pediatr Diabetes.

[19] Metsälä J., Lundqvist A., Virta L.J., Kaila M., Gissler M., Virtanen S.M. (2020). Use of antiasthmatic drugs and the risk of type 1 diabetes in children: a nationwide case–cohort study. Am J Epidemiol.

[20] Dharmage S.C., Perret J.L., Custovic A. (2019). Epidemiology of asthma in children and adults. Front Pediatr.

[21] Patterson C.C., Karuranga S., Salpea P., Saeedi P., Dahlquist G., Soltesz G. (2019). Worldwide estimates of incidence, prevalence and mortality of type 1 diabetes in children and adolescents: results from the International Diabetes Federation Diabetes Atlas, 9th edition. Diabetes Res Clin Pract.

[22] Okada H., Kuhn C., Feillet H., Bach J.F. (2010). The “hygiene hypothesis” for autoimmune and allergic diseases: an update. Clin Exp Immunol.

[23] Stene L.C., Nafstad P. (2001). Relation between occurrence of type 1 diabetes and asthma. Lancet.

[24] Thomsen S.F., Duffy D.L., Kyvik K.O., Skytthe A., Backer V. (2011). Relationship between type 1 diabetes and atopic diseases in a twin population. Allergy.

[25] Kyvik K.O., Green A., Beck-Nielsen H. (1995). Concordance rates of insulin dependent diabetes mellitus: a population based study of young Danish twins. BMJ.

[26] Ober C., Yao T.C. (2011). The genetics of asthma and allergic disease: a 21st century perspective. Immunol Rev.

[27] Zhou W., Kanai M., Wu K.H.H., Rasheed H., Tsuo K., Hirbo J.B. (2022). Global Biobank Meta-analysis Initiative: powering genetic discovery across human disease. Cell Genomics.

[28] Ferreira M.A.R., Mathur R., Vonk J.M., Szwajda A., Brumpton B., Granell R. (2019). Genetic architectures of childhood- and adult-onset asthma are partly distinct. Am J Hum Genet.

[29] Waage J., Standl M., Curtin J.A., Jessen L.E., Thorsen J., Tian C. (2018). Genome-wide association and HLA fine-mapping studies identify risk loci and genetic pathways underlying allergic rhinitis. Nat Genet.

[30] Sliz E., Huilaja L., Pasanen A., Laisk T., Reimann E., Mägi R. (2022). Uniting biobank resources reveals novel genetic pathways modulating susceptibility for atopic dermatitis. J Allergy Clin Immunol.

[31] Chiou J., Geusz R.J., Okino M.L., Han J.Y., Miller M., Melton R. (2021). Interpreting type 1 diabetes risk with genetics and single-cell epigenomics. Nature.

[32] Ferreira M.A., Vonk J.M., Baurecht H., Marenholz I., Tian C., Hoffman J.D. (2017). Shared genetic origin of asthma, hay fever and eczema elucidates allergic disease biology. Nat Genet.

[33] Li Y.R., Li J., Zhao S.D., Bradfield J.P., Mentch F.D., Maggadottir S.M. (2015). Meta-analysis of shared genetic architecture across ten pediatric autoimmune diseases. Nat Med.

[34] Lawlor D.A., Tilling K., Smith G.D. (2016). Triangulation in aetiological epidemiology. Int J Epidemiol.

[35] Ludvigsson J.F., Otterblad-Olausson P., Pettersson B.U., Ekbom A. (2009). The Swedish personal identity number: possibilities and pitfalls in healthcare and medical research. Eur J Epidemiol.

[36] Zagai U., Lichtenstein P., Pedersen N.L., Magnusson P.K.E. (2019). The Swedish Twin Registry: content and management as a research infrastructure. Twin Res Hum Genet.

[37] Magnusson P.K.E., Almqvist C., Rahman I., Ganna A., Viktorin A., Walum H. (2013). The Swedish Twin Registry: establishment of a biobank and other recent developments. Twin Res Hum Genet.

[38] Örtqvist A.K., Lundholm C., Wettermark B., Ludvigsson J.F., Ye W., Almqvist C. (2013). Validation of asthma and eczema in population-based Swedish drug and patient registers. Pharmacoepidemiol Drug Saf.

[39] Henriksen L., Simonsen J., Haerskjold A., Linder M., Kieler H., Thomsen S.F. (2015). Incidence rates of atopic dermatitis, asthma, and allergic rhinoconjunctivitis in Danish and Swedish children. J Allergy Clin Immunol.

[40] Rawshani A., Landin-Olsson M., Svensson A.M., Nyström L., Arnqvist H.J., Bolinder J. (2014). The incidence of diabetes among 0-34 year olds in Sweden: new data and better methods. Diabetologia.

[41] Lloyd-Jones L.R., Zeng J., Sidorenko J., Yengo L., Moser G., Kemper K.E. (2019). Improved polygenic prediction by Bayesian multiple regression on summary statistics. Nat Commun.

[42] Hudson J.I., Javaras K.N., Laird N.M., Vanderweele T.J., Pope H.G., Hernán M.A. (2008). A structural approach to the familial coaggregation of disorders. Epidemiology.

[43] Bulik-Sullivan B., Loh P.R., Finucane H.K., Ripke S., Yang J., Patterson N. (2015). LD score regression distinguishes confounding from polygenicity in genome-wide association studies. Nat Genet.

[44] Zheng J., Erzurumluoglu A.M., Elsworth B.L., Kemp J.P., Howe L., Haycock P.C. (2017). LD Hub: a centralized database and web interface to perform LD score regression that maximizes the potential of summary level GWAS data for SNP heritability and genetic correlation analysis. Bioinformatics.

[45] Rachmiel M., Bloch O., Shaul A.A., Ben-Yehudah G., Bistritzer Z., Weintrob N. (2011). Young patients with both type 1 diabetes mellitus and asthma have a unique IL-12 and IL-18 secretory pattern. Pediatr Diabetes.

[46] Rachmiel M., Bloch O., Bistritzer T., Weintrob N., Ofan R., Koren-Morag N. (2006). T_H_1/T_H_2 cytokine balance in patients with both type 1 diabetes mellitus and asthma. Cytokine.

[47] Klamt S., Vogel M., Kapellen T.M., Hiemisch A., Prenzel F., Zachariae S. (2015). Association between IgE-mediated allergies and diabetes mellitus type 1 in children and adolescents. Pediatr Diabetes.

[48] Hemminki K., Li X., Sundquist J., Sundquist K. (2009). Familial association between type 1 diabetes and other autoimmune and related diseases. Diabetologia.

[49] Kreiner E., Waage J., Standl M., Brix S., Pers T.H., Couto Alves A. (2017). Shared genetic variants suggest common pathways in allergy and autoimmune diseases. J Allergy Clin Immunol.

[50] Saleh N.M., Raj S.M., Smyth D.J., Wallace C., Howson J.M.M., Bell L. (2011). Genetic association analyses of atopic illness and proinflammatory cytokine genes with type 1 diabetes. Diabetes Metab Res Rev.

[51] Li X., Ampleford E.J., Howard T.D., Moore W.C., Torgerson D.G., Li H. (2012). Genome-wide association studies of asthma indicate opposite immunopathogenesis direction from autoimmune diseases. J Allergy Clin Immunol.

[52] Namjou B., Lape M., Malolepsza E., DeVore S.B., Weirauch M.T., Dikilitas O. (2022). Multiancestral polygenic risk score for pediatric asthma. J Allergy Clin Immunol.

[53] Demenais F., Margaritte-Jeannin P., Barnes K.C., Cookson W.O.C., Altmüller J., Ang W. (2018). Multiancestry association study identifies new asthma risk loci that colocalize with immune-cell enhancer marks. Nat Genet.

[54] Dijk F.N., Folkersma C., Gruzieva O., Kumar A., Wijga A.H., Gehring U. (2019). Genetic risk scores do not improve asthma prediction in childhood. J Allergy Clin Immunol.

[55] Sordillo J.E., Lutz S.M., Jorgenson E., Iribarren C., McGeachie M., Dahlin A. (2021). A polygenic risk score for asthma in a large racially diverse population. Clin Exp Allergy.

[56] Choi S.W., Mak T.S.H., O’Reilly P.F. (2020). Tutorial: a guide to performing polygenic risk score analyses. Nat Protoc.

