# Supplementary material for: Disentangling the comorbidity between allergic disease and type 1 diabetes using genetically informative designs
Source: J Allergy Clin Immunol Glob. 2025 Jun 23;4(4):100519. doi: 10.1016/j.jacig.2025.100519 (PMC12305212; doi:10.1016/j.jacig.2025.100519)
Supplement: Supplementary Figs and Tables [file mmc1.docx]

Smew et al. *Disentangling the comorbidity between allergic disease and type 1 diabetes using genetically informative designs*

**Supplement**

**Table of Contents**

[A. Supplementary Methods 2](#_Toc188727427)

[B. Supplementary Figures 4](#_Toc188727428)

[Figure S1. Validation of polygenic risk scores 4](#_Toc188727429)

[C. Supplementary Tables 5](#_Toc188727430)

[Table S1. Overview of nationwide Swedish population registers 5](#_Toc188727431)

[Table S2. Register-based definitions of allergic diseases and type 1 diabetes 6](#_Toc188727432)

[Table S3. Overview of included genome-wide association studies 7](#_Toc188727433)

[Table S4. Genetic and environmental sharing across relatives 8](#_Toc188727434)

[Table S5. Association between asthma and type 1 diabetes within individuals and the familial co-aggregation between relatives 9](#_Toc188727435)

[Table S6. Association between allergic rhinitis and type 1 diabetes within individuals and the familial co-aggregation between relatives 10](#_Toc188727436)

[Table S7. Association between eczema and type 1 diabetes within individuals and the familial co-aggregation between relatives 11](#_Toc188727437)

[Table S8. Sensitivity analysis of within-individual associations between allergic diseases and type 1 diabetes 12](#_Toc188727438)

[Table S9. Study characteristics of parents within the study population (born 1987–2017), and of parents to individuals in the study population irrespective of parental birth year 13](#_Toc188727439)

[Table S10. Sensitivity analysis of familial co-aggregation between allergic diseases and type 1 diabetes among all possible parent-offspring pairs regardless of parental birth year 14](#_Toc188727440)

[Table S11. Exploratory post-hoc analysis of within-individual associations between allergic diseases and type 1 diabetes 15](#_Toc188727441)

[Table S12. Univariate (within disease) estimates for allergic diseases and type 1 diabetes separately, presented as concordance rates and tetrachoric correlations in subsets of same-generation relatives 16](#_Toc188727442)

[Table S13. Bivariate (between diseases) estimates for the comorbidity between allergic diseases and type 1 diabetes, presented as concordance rates and tetrachoric correlations in the whole population and among subsets of same-generation relatives 17](#_Toc188727443)

[D. References 18](#_Toc188727444)

# A. Supplementary Methods

**Genotyped data**

For the polygenic risk score part of this study, we included in our genotyped cohort (Figure 1B) all unique individuals from the twin studies “Study of Twin Adults: Genes and Environment” (STAGE), “Young Adult Twins in Sweden Study” (YATSS) and "Child and Adolescent Twin Study of Sweden” (CATSS) that had provided DNA samples for genotyping (n=31,089). For CATSS DNA samples from saliva were analysed in two rounds, using the Illumina PsychArray BeadChip for the first and the 650K Illumina Global Screening Array (GSA) BeadChip for the second. The latter method was also used to genotype DNA samples from blood provided from STAGE and YATSS participants. Genotyped data underwent standard quality control procedures which included removing low quality samples (for instance due to contamination), those with non-European ancestry or missing principal components. We also imputed missing data among monozygotic twins if they had a genotyped twin. In total, 30,880 samples were used in the analysis.

**Polygenic risk scores**

Polygenic risk scores for each trait (asthma, allergic rhinitis, eczema, type 1 diabetes) were generated based on summary statistics publicly available from the to-date largest genome-wide association studies (GWAS) of each respective disease (discovery sets) and applied on aforementioned genotyped data treating individuals from each respective twin study (STAGE, YATSS, CATSS) as separate targets sets. We searched the NHGRI-EBI GWAS Catalog (www.ebi.ac.uk/gwas), Medline, MedRxiv and UK Biobank for summary statistics from GWAS up until January, 2022. The original data provided from each respective publication (Table S3) was downloaded. GWAS to then use were identified based on sample size, European ancestry and not including the Swedish twins in our targets sets. PRS were calculated separately for each twin study (STAGE, YATSS, CATSS) from which individuals in the cohort originated, standardised and then combined. To generate PRS the SBayesR method was applied, described elsewhere (1). However, given that for several of our traits significant variants in the major histocompatibility complex (MHC) region have been identified and that SBayesR excludes the MHC region, we added back in the most significant SNP in the MHC region variants after applying SBayesR. PRS were generated using “plink2” and standardised.

To validate the generated PRS before applying them in our main regression analysis (PRS for one disease predicting the other disease) we first correlated the disease-PRS to the same disease phenotype. From logistic regression models adjusted for population stratification and accounting for familial clustering using robust standard errors, odds ratios by decile (allergic diseases) or quartile (type 1 diabetes) were estimated and displayed in forest plots (Figure S1). Area Under the receiver operating Curves (AUC) were presented as a measure of PRS predication performance.

# B. Supplementary Figures

Figure S1. Validation of polygenic risk scores presented as the association between deciles (A, B, C) or quantiles (D) of polygenic risk scores for a trait and the phenotypic measure of the same trait, with the lowest group as reference. A, asthma. B, allergic rhinitis. C, eczema. D, type 1 diabetes. The area under the curve (AUC) is also presented, representing the quality of prediction. PRS for asthma, eczema and type 1 diabetes seemed to better predict the phenotype than allergic rhinitis. AUC was best for type 1 diabetes (0.79)

# C. Supplementary Tables

## Table S1. Overview of nationwide Swedish population registers

| **Register** | **Held by** | **Description** | **In this study** | **References** |
| --- | --- | --- | --- | --- |
|  |  |  |  |  |
| Total Population Register | Statistics Sweden | Sociodemographic information including 100% of births in Sweden, death, civil status, migration and family relationships. Started in 1968 | Data on birth, death, and migration | (2) |
|  |  |  |  |  |
|  |  |  |  |  |
| Multi-generation Register | Statistics Sweden | A part of the Total Population Register. Data on biological parents for individuals born 1932 or onwards and registered as living in Sweden in 1960 or onwards | Identification of subsets of relatives | (3) |
|  |  |  |  |  |
|  |  |  |  |  |
| National Patient Register | National Board of Health and Welfare | Data on in-patient care since 1973, with full coverage since 1987, and >80% out-patient care since 2001 with diagnoses classified according to ICD | Disease definitions for allergic diseases and type 1 diabetes | (4) |
|  |  |  |  |  |
|  |  |  |  |  |
| Prescribed Drug Register | National Board of Health and Welfare | Data on all dispensed medication prescriptions according to ATC since 1 July 2005 | Disease definitions for allergic diseases and type 1 diabetes | (5) |
|  |  |  |  |  |
| Swedish Twin Registry | Steering committee at Karolinska Institutet | Data on almost all twins in Sweden since 1886, collected in several twin cohort studies. Information differs depending on twin cohort but includes questionnaires and biological samples | Zygosity,  genotyped information for polygenic risk scores | (6,7) |
|  |  |  |  |  |

Abbreviations: ATC, Anatomical Therapeutic Chemical classification. ICD, International Classification of Diseases

## Table S2. Register-based definitions of allergic diseases and type 1 diabetes

| **Disease** | **Definition** | **Ref** |
| --- | --- | --- |
|  |  |  |
| Asthma | **<4.5 years of age**: Medication criteria AND any diagnosis (ICD-10 J45/J46) ever  **≥4.5 years of age**: Medication criteria OR any diagnosis (ICD-10 J45/J46) ever  **Medication criteria**:  **A**. ≥2 dispenses of inhaled corticosteroids (ATC R03BA)/leukotriene receptor antagonists (ATC R03DC)/fixed beta-2-agonist + corticosteroid combinations  (ATC R03AK), with 2 weeks between dispenses in children <4.5 years  OR  **B**. ≥3 dispenses of short acting beta-2-agonists (ATC R03AC) within 12 months | (8) |
|  |  |  |
|  |  |  |
| Allergic rhinitis | Either of:  **A**. ≥1 record of allergic rhinitis diagnosis (ICD-10 J30/J31.0)  **B**. ≥2 dispenses of nasal corticosteroids (ATC R01AD), excluding individuals with diagnoses for nasal polyps, acute or chronic sinusitis (ICD-10 J33, J01, J32)  **C**. ≥2 dispenses of oral antihistamines (ATC R06A), excluding individuals with diagnoses for pruritus or urticaria (ICD-10 L29, L50)  **D**. ≥1 dispense of allergen-specific immunotherapy (ATC V01A) or eyedrops for allergic conjunctivitis (ATC S01GX) | (9) |
|  |  |  |
|  |  |  |
| Eczema | Either of:  **A**. ≥1 record of eczema diagnosis (ICD-10 L20, L308C)  **B**. ≥1 dispense of specialised agents for dermatitis (ATC D11AH)  **C**. ≥2 dispenses of topical corticosteroids (ATC D07)  Without having any exclusion criteria including diagnoses for other forms of dermatitis or dispensed medication for psoriasis or skin infections. Full list of exclusion criteria in Appendix of Henriksen et al. | (9) |
|  |  |  |
|  |  |  |
| Type 1 diabetes | Either of:  **A**. Diagnosis of type 1 diabetes before 18 years of age (ICD-10 E10)  **B**. ≥1 dispense of insulin (ATC A10A) before 18 years of age for those born 1987­–2020, and at any age but without any oral antidiabetics (ATC A10B–X) for older individuals due to register coverage with the Prescribed Drug Register starting in 2005 | (10) |
|  |  |  |

## Table S3. Overview of included genome-wide association studies

| **Disease** | **Phenotype definition** | **Sample size** | **Origin** | **Reference** |
| --- | --- | --- | --- | --- |
|  |  |  |  |  |
| Asthma | Childhood-onset,  adult-onset | 13,962 cases of childhood-onset, 26,582 cases of adult-onset, 300,671 common controls | UK Biobank | Ferreira et al, American Journal of Human Genetics, 2019 (11) |
|  |  |  |  |  |
|  |  |  |  |  |
| Asthma | Any history of asthma | 153,763 cases, 1,647,022 controls | Meta-analysis including European ancestry samples from 14 biobanks | Zhou et al, Cell Genomics 2022 (12) |
|  |  |  |  |  |
|  |  |  |  |  |
| Allergic rhinitis | Either a diagnosis or symptoms of allergic rhinitis | 59,762 cases, 152,358 controls | Meta-analysis including 23andMe, UK Biobank and, deCODE being the three largest studies | Waage et al, Nature Genetics 2018 (13) |
|  |  |  |  |  |
|  |  |  |  |  |
| Eczema | Diagnostic records of any of the following relevant ICD-codes for eczema/atopic dermatitis: ICD-10 L20; ICD-9 6918; ICD-8 691 | 22,474 cases, 774,187 controls | Meta-analysis including European ancestry individuals from FinnGen, Estonian Biobank and UK Biobank | Sliz et al, Journal of Allergy and Clinical Immunology (14) |
|  |  |  |  |  |
|  |  |  |  |  |
| Type 1 diabetes | For non-UK Biobank samples, cases of type 2 diabetes were excluded. For UK Biobank samples, definition based on type 1 diabetes diagnosis or insulin treatment within a year of diagnosis, as well as excluding type 2 diabetes diagnosis | 18,942 cases, 501,638 control | Meta-analysis of European ancestry individuals from multiple sources including, among others, the Type 1 Diabetes Genetics Consortium, UK Biobank and FinnGen | Chiou et al, Nature 2021 (15) |
|  |  |  |  |  |

## Table S4. Genetic and environmental sharing across relatives

| **Relative** | **Degree of shared segregating genes** | **Environmental sharing** | **Interpretation of familial  co-aggregation** |
| --- | --- | --- | --- |
|  |  |  |  |
| Parent-offspring | 50% additive, 0% dominant | Family environment constant across generations | Positive associations represent shared genetic or environmental sharing |
|  |  |  |  |
|  |  |  |  |
| Monozygotic twins | 100% additive, 100% dominant | 100% pregnancy and childhood | Higher estimates in monozygotic compared to dizygotic twins suggest genetic contribution |
|  |  |  |  |
|  |  |  |  |
| Dizygotic twins | 50% additive, 25% dominant | 100% pregnancy and childhood | Higher estimates in dizygotic twins than full siblings suggest pregnancy-specific environmental factors |
|  |  |  |  |
|  |  |  |  |
| Full siblings | 50% additive, 25% dominant | <100% pregnancy: factors that are constant across pregnancies, childhood | Higher estimates in full siblings than maternal half-siblings suggest genetic contribution |
|  |  |  |  |
|  |  |  |  |
| Maternal  half-siblings | 25% additive, 0% dominant | <100% pregnancy: factors that are constant across pregnancies, 100% early childhood, though potentially less | Higher estimates in maternal than paternal half-siblings suggest pregnancy-related or other environmental factors |
|  |  |  |  |
|  |  |  |  |
| Paternal  half-siblings | 25% additive, 0% dominant | 0% pregnancy, <100% childhood since children tend to live with their mother after separation, especially early in life |  |
|  |  |  |  |

## Table S5. Association between asthma and type 1 diabetes within individuals and the familial co-aggregation between relatives

|  | | **Asthma** | |  | **No asthma** | |  | **Odds ratio (95% CI)** | | | | | | | | |
| --- | --- | --- | --- | --- | --- | --- | --- | --- | --- | --- | --- | --- | --- | --- | --- | --- |
|  | | Total | Type 1 diabetes (%) |  | Total | Type 1  diabetes (%) |  | Crude | | | Adjusted for sex and birth year | | | Additionally adjusted for relative's asthma | | |
|  | |  |  |  |  |  |  |  | | |  | | |  | | |
| Within-individual | | 475,422 | 3,259 (0.69) |  | 2,723,820 | 16,876 (0.62) |  | 1.11 (1.07, 1.15) | | | 1.11 (1.07, 1.15) | | | - | | |
|  | |  |  | |  |  | |  | | |  | | |  | | |
| **Familial co-aggregation** | | |  | |  |  | |  | | |  | | |  | | |
|  | |  |  |  |  |  |  | |  | | |  | | |  | |
| Parent-offspring | | 43,581 | 329 (0.75) |  | 240,488 | 1,502 (0.62) |  | | 1.21 (1.07, 1.38) | | | 1.21 (1.06, 1.38) | | | 1.20 (1.06, 1.37) | |
|  | |  |  |  |  |  |  | |  | | |  | | |  | |
|  | *Mother-offspring* | 27,569 | 2017 (0.75) |  | 151,639 | 915 (0.60) |  | | | 1.25 (1.06, 1.47) | | | 1.25 (1.06, 1.47) | | | 1.24 (1.05, 1.45) |
|  |  |  |  |  |  |  |  | | |  | | |  | | |  |
|  | *Father-offspring* | 16,012 | 122 (0.76) |  | 88,849 | 587 (0.66) |  | | | 1.15 (0.94, 1.42) | | | 1.15 (0.94, 1.41) | | | 1.15 (0.93, 1.42) |
|  |  |  |  |  |  |  |  | | |  | | |  | | |  |
| Monozygotic twins | | 2,322 | 17 (0.73) |  | 12,592 | 62 (0.49) |  | | | 1.49 (0.84, 2.64) | | | 1.51 (0.85, 2.69) | | | 1.62 (0.88, 3.01) |
|  | |  |  |  |  |  |  | | |  | | |  | | |  |
| Dizygotic twins | | 6,985 | 42 (0.60) |  | 36,213 | 216 (0.60) |  | | | 1.00 (0.72, 1.41) | | | 1.03 (0.74, 1.44) | | | 1.04 (0.74, 1.45) |
|  | |  |  |  |  |  |  | | |  | | |  | | |  |
| Full siblings | | 503,355 | 3,582 (0.71) |  | 2,990,313 | 18,911 (0.63) |  | | | 1.13 (1.08, 1.17) | | | 1.14 (1.09, 1.18) | | | 1.12 (1.08, 1.17) |
|  | |  |  |  |  |  |  | | |  | | |  | | |  |
| Maternal half-siblings | | 97,747 | 657 (0.67) |  | 484,705 | 3,281 (0.68) |  | | | 0.99 (0.90, 1.09) | | | 1.00 (0.91, 1.09) | | | 0.98 (0.89, 1.08) |
|  | |  |  |  |  |  |  | | |  | | |  | | |  |
| Paternal half-siblings | | 108,056 | 732 (0.68) |  | 550,754 | 3,610 (0.66) |  | | | 1.03 (0.95, 1.13) | | | 1.04 (0.95, 1.13) | | | 1.03 (0.94, 1.12) |
|  | |  |  |  |  |  |  | | |  | | |  | | |  |

## Table S6. Association between allergic rhinitis and type 1 diabetes within individuals and the familial co-aggregation between relatives

|  | **Allergic rhinitis** | |  | **No allergic rhinitis** | |  | **Odds ratio (95% CI)** | | | | | |
| --- | --- | --- | --- | --- | --- | --- | --- | --- | --- | --- | --- | --- |
|  | Total | Type 1 diabetes (%) |  | Total | Type 1 diabetes (%) |  | Crude | | Adjusted for sex and birth year | | Additionally adjusted for relative's allergic rhinitis | |
|  |  |  |  |  |  |  |  | |  | |  | |
| Within-individual | 924,023 | 6,960 (0.75) |  | 2,275,219 | 13,175 (0.58) |  | 1.30 (1.27, 1.34) | | 1.23 (1.19, 1.27) | | - | |
|  |  |  | |  |  | |  | |  | |  | |
| **Familial co-aggregation** | |  | |  |  | |  | |  | |  | |
|  | |  | |  |  | |  | |  | |  | |
| Parent-offspring | 49,538 | 357 (0.72) |  | 234,531 | 1,474 (0.63) |  | | 1.15 (1.02, 1.29) | | 1.15 (1.02, 1.30) | | 1.14 (1.01, 1.28) |
|  |  |  |  |  |  |  | |  | |  | |  |
| *Mother-offspring* | 31,746 | 219 (0.69) |  | 147,462 | 903 (0.61) |  | | 1.13 (0.97, 1.32) | | 1.13 (0.97, 1.32) | | 1.10 (0.95, 1.29) |
|  |  |  |  |  |  |  | |  | |  | |  |
| *Father-offspring* | 17,792 | 138 (0.78) |  | 87,069 | 571 (0.66) |  | | 1.18 (0.98, 1.43) | | 1.19 (0.99, 1.44) | | 1.19 (0.98, 1.44) |
|  |  |  |  |  |  |  | |  | |  | |  |
| Monozygotic twins | 4,092 | 27 (0.66) |  | 10,822 | 52 (0.48) |  | | 1.38 (0.81, 2.34) | | 1.37 (0.81, 2.31) | | 1.29 (0.80, 2.10) |
|  |  |  |  |  |  |  | |  | |  | |  |
| Dizygotic twins | 11,798 | 70 (0.59) |  | 31,400 | 188 (0.60) |  | | 0.99 (0.75, 1.31) | | 0.93 (0.71, 1.24) | | 0.92 (0.70, 1.21) |
|  |  |  |  |  |  |  | |  | |  | |  |
| Full siblings | 978,702 | 6,782 (0.69) |  | 2,514,966 | 15,711 (0.62) |  | | 1.11 (1.08, 1.15) | | 1.07 (1.03, 1.10) | | 1.04 (1.01, 1.08) |
|  |  |  |  |  |  |  | |  | |  | |  |
| Maternal half-siblings | 183,324 | 1,295 (0.71) |  | 399,128 | 2,643 (0.66) |  | | 1.07 (0.99, 1.15) | | 1.06 (0.98, 1.14) | | 1.04 (0.97, 1.12) |
|  |  |  |  |  |  |  | |  | |  | |  |
| Paternal half-siblings | 209,681 | 1,381 (0.66) |  | 449,129 | 2,961 (0.66) |  | | 1.00 (0.93, 1.07) | | 1.00 (0.93, 1.07) | | 0.99 (0.92, 1.06) |
|  |  |  |  |  |  |  | |  | |  | |  |

## Table S7. Association between eczema and type 1 diabetes within individuals and the familial co-aggregation between relatives

|  | **Eczema** | |  | **No eczema** | |  | **Odds ratio (95% CI)** | | |
| --- | --- | --- | --- | --- | --- | --- | --- | --- | --- |
|  | Total | Type 1 diabetes (%) |  | Total | Type 1 diabetes (%) |  | Crude | Adjusted for sex and birth year | Additionally adjusted for relative's asthma |
|  |  |  |  |  |  |  |  |  |  |
| Within-individual | 516,993 | 3,963 (0.77) |  | 2,682,249 | 16,172 (0.60) |  | 1.27 (1.23, 1.32) | 1.31 (1.26, 1.35) | - |
|  |  |  | |  |  | |  |  |  |
| **Familial co-aggregation** | |  | |  |  | |  |  |  |
|  | |  | |  |  | |  |  |  |
| Parent-offspring | 41,646 | 270 (0.65) |  | 242,423 | 1,561 (0.64) |  | 1.01 (0.88, 1.15) | 1.01 (0.88, 1.15) | 1.01 (0.88, 1.14) |
|  |  |  |  |  |  |  |  |  |  |
| *Mother-offspring* | 26,408 | 162 (0.61) |  | 152,800 | 960 (0.63) |  | 0.98 (0.83, 1.15) | 0.98 (0.83, 1.15) | 0.98 (0.83, 1.15) |
|  |  |  |  |  |  |  |  |  |  |
| *Father-offspring* | 15,238 | 108 (0.71) |  | 89,623 | 601 (0.67) |  | 1.06 (0.86, 1.31) | 1.06 (0.86, 1.31) | 1.05 (0.85, 1.30) |
|  |  |  |  |  |  |  |  |  |  |
| Monozygotic twins | 1,991 | 6 (0.30) |  | 12,923 | 73 (0.56) |  | 0.53 (0.24, 1.20) | 0.54 (0.24, 1.21) | 0.52 (0.23, 1.20) |
|  |  |  |  |  |  |  |  |  |  |
| Dizygotic twins | 6,343 | 42 (0.66) |  | 36,855 | 216 (0.59) |  | 1.13 (0.81, 1.57) | 1.14 (0.82, 1.59) | 1.08 (0.78, 1.50) |
|  |  |  |  |  |  |  |  |  |  |
| Full siblings | 558,320 | 3,597 (0.64) |  | 2,935,348 | 18,896 (0.64) |  | 1.00 (0.96, 1.04) | 1.02 (0.98, 1.06) | 0.99 (0.95, 1.03) |
|  |  |  |  |  |  |  |  |  |  |
| Maternal half-siblings | 92,621 | 654 (0.71) |  | 489,831 | 3,284 (0.67) |  | 1.05 (0.96, 1.16) | 1.06 (0.96, 1.17) | 1.04 (0.95, 1.15) |
|  |  |  |  |  |  |  |  |  |  |
| Paternal half-siblings | 109,924 | 756 (0.69) |  | 548,886 | 3,586 (0.65) |  | 1.04 (0.97, 1.15) | 1.06 (0.97, 1.15) | 1.05 (0.96, 1.14) |
|  |  |  |  |  |  |  |  |  |  |

## Table S8. Sensitivity analysis of within-individual associations between allergic diseases and type 1 diabetes

|  | **Allergic disease** | |  | **No allergic disease** | |  | **Odds ratio (95% CI)** | |
| --- | --- | --- | --- | --- | --- | --- | --- | --- |
|  | Total | Type 1 diabetes (%) |  | Total | Type 1 diabetes (%) |  | Crude | Adjusted for sex and birth year |
|  |  |  |  |  |  |  |  |  |
| **Minimum 10 years of follow-up (born 1987–2011)**  n=2,599,653 |  |  |  |  |  |  |  |  |
|  |  |  |  |  |  |  |  |  |
| Asthma | 397,355 | 3,040 (0.77) |  | 2,202,298 | 15,783 (0.72) |  | 1.07 (1.03, 1.11) | 1.07 (1.03, 1.11) |
|  |  |  |  |  |  |  |  |  |
| Allergic rhinitis | 831,594 | 6,679 (0.80) |  | 1,768,059 | 12,144 (0.69) |  | 1.17 (1.14, 1.21) | 1.17 (1.14, 1.21) |
|  |  |  |  |  |  |  |  |  |
| Eczema | 425,401 | 3,614 (0.85) |  | 2,174,252 | 15,209 (0.70) |  | 1.22 (1.17, 1.26) | 1.23 (1.19, 1.28) |
|  |  |  |  |  |  |  |  |  |
|  |  |  |  |  |  |  |  |  |
| **Exclusion of individuals  who died or emigrated  during follow-up**  n=3,097,346 |  |  |  |  |  |  |  |  |
|  |  |  |  |  |  |  |  |  |
| Asthma | 469,005 | 3,227 (0.69) |  | 2,628,341 | 16,582 (0.63) |  | 1.09 (1.05, 1.13) | 1.09 (1.05, 1.14) |
|  |  |  |  |  |  |  |  |  |
| Allergic rhinitis | 907,263 | 6,859 (0.76) |  | 2,190,083 | 12,950 (0.59) |  | 1.28 (1.24, 1.32) | 1.20 (1.16, 1.24) |
|  |  |  |  |  |  |  |  |  |
| Eczema | 509,368 | 3,962 (0.78) |  | 2,587,978 | 15,847 (0.61) |  | 1.27 (1.23, 1.32) | 1.30 (1.26, 1.35) |
|  |  |  |  |  |  |  |  |  |

## Table S9. Study characteristics of parents within the study population (born 1987–2017), and of parents to individuals in the study population irrespective of parental birth year

|  | | **Parents within the study population**  **born 1987–2017** | | | **Parents to individuals in study population irrespective of parental birth year** | | |
| --- | --- | --- | --- | --- | --- | --- | --- |
| n (%) | | Any parent | Mothers | Fathers | Any parent | Mothers | Fathers |
| Total number of unique individuals | | 192,801 | 118,769 | 74,302 | 3,358,610 | 1,680,141 | 1,678,469 |
| Number of parent-offspring pairs in analysis | | 284,069 | 179,208 | 104,861 | 6,398,484 | 3,199,254 | 3,199,230 |
|  | |  |  |  |  |  |  |
| Sex | |  |  |  |  |  |  |
|  | *Male* | 104,861 (36.9) | - | - | 3,199,230 (50.0) | - | - |
|  | *Female* | 179,208 (63.1) | - | - | 3,199,254 (50.0) | - | - |
|  | |  |  |  |  |  |  |
| Birth year, mean  (min–max, SD) | | 1989.3 (1987–2003, 2.2) | 1989.5 (1987–2003, 2.6) | 1989.2 (1987–2002, 2.1) | 1970.5 (1911–2003, 10.2) | 1972.0 (1935–2003, 9.8) | 1969.0 (1911–2002, 10.3) |
|  | |  |  |  |  |  |  |
| Asthma | | 41,360 (14.56) | 28,367 (15.9) | 12,993 (12.4) | - | - | - |
|  | *Age at onset, y (SD)* | 18.0 (7.4) | 18.4 (7.1) | 17.1 (7.9) | - | - | - |
|  |  |  |  |  |  |  |  |
| Allergic rhinitis | | 115,096 (40.52) | 84,713 (47.3) | 30,383 (29.0) | - | - | - |
|  | *Age at onset, y (SD)* | 20.9 (5.2) | 21.0 (5.0) | 20.6 (5.6) | - | - | - |
|  |  |  |  |  |  |  |  |
| Eczema | | 40,030 (14.1) | 29,617 (16.5) | 10,413 (9.9) | - | - | - |
|  | *Age at onset, y (SD)* | 20.2 (5.6) | 20.0 (5.5) | 20.8 (5.9) | - | - | - |
|  |  |  |  |  |  |  |  |
| Type 1 diabetes | | 1,831 (0.6) | 1,122 (0.6) | 709 (0.7) | 75,690 (1.2) | 27,522 (0.9) | 48,168 (1.5) |
|  | *Age at onset, y (SD)* | 10.5 (4.3) | 10.2 (4.3) | 10.9 (4.3) | - | - | - |
|  | |  |  |  |  |  |  |
| Comorbid asthma  and type 1 diabetes | | 282 (0.10) | 191 (0.1) | 91 (0.1) | - | - | - |
| Comorbid allergic rhinitis and type 1 diabetes | | 797 (0.28) | 581 (0.3) | 216 (0.2) | - | - | - |
| Comorbid eczema  and type 1 diabetes | | 273 (0.10) | 188 (0.1) | 85 (0.1) | - | - | - |

## Table S10. Sensitivity analysis of familial co-aggregation between allergic diseases and type 1 diabetes among all possible parent-offspring pairs regardless of parental birth year

|  | | **Allergic disease** | |  | **No allergic disease** | |  | **Odds ratio (95% CI)** | |
| --- | --- | --- | --- | --- | --- | --- | --- | --- | --- |
|  | | Total | Parental type 1 diabetes (%) |  | Total | Parental type 1 diabetes (%) |  | Crude | Adjusted for sex and birth year |
|  | |  |  |  |  |  |  |  |  |
| **Asthma** | |  |  |  |  |  |  |  |  |
| Parent-offspring | | 950,844 | 12,147 (1.28) |  | 5,447,640 | 63,543 (1.17) |  | 1.10 (1.07, 1.12) | 1.11 (1.09, 1.13) |
|  | |  |  |  |  |  |  |  |  |
|  | *Mother-offspring* | 475,418 | 4,658 (0.98) |  | 2,723,810 | 22,864 (0.84) |  | 1.17 (1.13, 1.21) | 1.17 (1.13, 1.21) |
|  |  |  |  |  |  |  |  |  |  |
|  | *Father-offspring* | 471,176 | 7,489 (1.59) |  | 2,692,653 | 40,679 (1.51) |  | 1.05 (1.02, 1.08) | 1.08 (1.05, 1.10) |
|  | |  |  |  |  |  |  |  |  |
|  | |  |  |  |  |  |  |  |  |
| **Allergic rhinitis** | |  |  |  |  |  |  |  |  |
| Parent-offspring | | 1,848,046 | 23,691 (1.28) |  | 4,550,438 | 51,999 (1.14) |  | 1.12 (1.10, 1.14) | 1.06 (1.04, 1.07) |
|  | |  |  |  |  |  |  |  |  |
|  | *Mother-offspring* | 924,021 | 8,562 (0.93) |  | 2,275,207 | 18,960 (0.83) |  | 1.11 (1.08, 1.14) | 1.09 (1.06, 1.12) |
|  |  |  |  |  |  |  |  |  |  |
|  | *Father-offspring* | 916,032 | 15,129 (1.65) |  | 2,247,797 | 33,039 (1.47) |  | 1.13 (1.10, 1.15) | 1.04 (1.02, 1.06) |
|  | |  |  |  |  |  |  |  |  |
|  | |  |  |  |  |  |  |  |  |
| **Eczema** | |  |  |  |  |  |  |  |  |
| Parent-offspring | | 1,033,986 | 12,330 (1.19) |  | 5,364,498 | 63,360 (1.18) |  | 1.01 (0.99, 1.03) | 1.03 (1.00, 1.05) |
|  | |  |  |  |  |  |  |  |  |
|  | *Mother-offspring* | 516,990 | 4,623 (0.89) |  | 2,682,238 | 22,899 (0.85) |  | 1.05 (1.01, 1.08) | 1.05 (1.02, 1.09) |
|  |  |  |  |  |  |  |  |  |  |
|  | *Father-offspring* | 509,775 | 7,707 (1.51) |  | 2,654,054 | 40,461 (1.52) |  | 0.99 (0.97, 1.02) | 1.01 (0.98, 1.04) |
|  |  |  |  |  |  |  |  |  |  |

## Table S11. Exploratory post-hoc analysis of within-individual associations between allergic diseases and type 1 diabetes

|  | **Allergic disease** | |  | **No allergic disease** | |  | **Odds ratio (95% CI)** | |
| --- | --- | --- | --- | --- | --- | --- | --- | --- |
|  | Total | Type 1 diabetes (%) |  | Total | Type 1 diabetes (%) |  | Crude | Adjusted for sex and birth year |
|  |  |  |  |  |  |  |  |  |
| **Alternative definitions of allergic disease** |  |  |  |  |  |  |  |  |
|  |  |  |  |  |  |  |  |  |
| Asthma stratified by phenotype |  |  |  |  |  |  |  |  |
| *Non-allergic asthma  (only asthma)* | 160,794 | 968 (0.60) |  | 2,787,725 | 17,216 (0.62) |  | 0.97 (0.91, 1.04) | 1.03 (0.96, 1.10) |
| *Allergic asthma  (asthma and at least one of allergic rhinitis/eczema)* | 324,495 | 2,353 (0.73) |  | 2,787,725 | 17,216 (0.62) |  | 1.18 (1.13, 1.23) | 1.15 (1.10, 1.20) |
|  |  |  |  |  |  |  |  |  |

## Table S12. Univariate (within disease) estimates for allergic diseases and type 1 diabetes separately, presented as concordance rates and tetrachoric correlations in subsets of same-generation relatives

|  | **Monozygotic twins** | **Dizygotic twins** | **Full siblings** | **Maternal  half-siblings** | **Paternal  half-siblings** |
| --- | --- | --- | --- | --- | --- |
|  |  |  |  |  |  |
| **Asthma** |  |  |  |  |  |
| Number of concordant pairs | 1,276 | 2,040 | 138,650 | 24,496 | 22,380 |
|  |  |  |  |  |  |
| Concordance rate^*^ (95% CI) | 0.55 (0.53, 0.57) | 0.29 (0.28, 0.30) | 0.28 (0.27, 0.28) | 0.25 (0.25, 0.25) | 0.21 (0.20, 0.21) |
|  |  |  |  |  |  |
| Intraclass correlation**^†^** (95% CI) | 0.73 (0.71, 0.75) | 0.30 (0.29, 0.32) | 0.31 (0.31, 0.31) | 0.20 (0.20, 0.21) | 0.11 (0.10, 0.12) |
|  |  |  |  |  |  |
| **Allergic rhinitis** |  |  |  |  |  |
| Number of concordant pairs | 2,072 | 4,450 | 366,648 | 66,910 | 71,476 |
|  |  |  |  |  |  |
| Concordance rate^*^ (95% CI) | 0.51 (0.49, 0.52) | 0.38 (0.37, 0.39) | 0.37 (0.37, 0.38) | 0.36 (0.36, 0.37) | 0.34 (0.34, 0.34) |
|  |  |  |  |  |  |
| Intraclass correlation**^†^** (95% CI) | 0.51 (0.49, 0.53) | 0.24 (0.23, 0.26) | 0.23 (0.22, 0.23) | 0.12 (0.12, 0.13) | 0.06 (0.05, 0.06) |
|  |  |  |  |  |  |
| **Eczema** |  |  |  |  |  |
| Number of concordant pairs | 748 | 1,452 | 132,858 | 18,158 | 20,948 |
|  |  |  |  |  |  |
| Concordance rate^*^ (95% CI) | 0.38 (0.35, 0.40) | 0.23 (0.22, 0.24) | 0.24 (0.24, 0.24) | 0.20 (0.19, 0.20) | 0.19 (0.19, 0.19) |
|  |  |  |  |  |  |
| Intraclass correlation**^†^** (95% CI) | 0.52 (0.49, 0.55) | 0.20 (0.18, 0.23) | 0.19 (0.19, 0.19) | 0.10 (0.09, 0.10) | 0.06 (0.06, 0.07) |
|  |  |  |  |  |  |
| **Type 1 diabetes** |  |  |  |  |  |
| Number of concordant pairs | 30 | 16 | 1672 | 102 | 94 |
|  |  |  |  |  |  |
| Concordance rate^*^ (95% CI) | 0.38 (0.27, 0.50) | 0.06 (0.04, 0.10) | 0.07 (0.07, 0.08) | 0.03 (0.02, 0.03) | 0.02 (0.02, 0.03) |
|  |  |  |  |  |  |
| Intraclass correlation**^†^** (95% CI) | 0.83 (0.77, 0.89) | 0.38 (0.28, 0.48) | 0.41 (0.40, 0.42) | 0.20 (0.17, 0.23) | 0.17 (0.14, 0.21) |
|  |  |  |  |  |  |

^*^The concordance rate (univariate) is the proportion of individuals with one disease if the relative had the same disease

^†^The intraclass correlation is the correlation within the same diseases, between individuals

## Table S13. Bivariate (between diseases) estimates for the comorbidity between allergic diseases and type 1 diabetes, presented as concordance rates and tetrachoric correlations in the whole population and among subsets of same-generation relatives

|  | **Whole population** | **Monozygotic twins** | **Dizygotic twins** | **Full siblings** | **Maternal  half-siblings** | **Paternal  half-siblings** |
| --- | --- | --- | --- | --- | --- | --- |
|  |  |  |  |  |  |  |
| **Asthma and type 1 diabetes** |  |  |  |  |  |  |
| Number of concordant pairs | - | 17 | 42 | 3,582 | 657 | 732 |
| Concordance rate**^*^**  (95% CI) | - | 0.01  (0.00, 0.01) | 0.01  (0.00, 0.01) | 0.01  (0.01, 0.01) | 0.01  (0.01, 0.01) | 0.01  (0.01, 0.01) |
| Phenotypic correlation**^†^**  (95% CI) | 0.02  (0.01, 0.03) | 0.01  (-0.10, 0.13) | -0.01  (-0.07, 0.06) | 0.02  (0.01, 0.03) | 0.02  (0.01, 0.04) | 0.03  (0.02, 0.05) |
| Cross-relative cross-trait correlation^‡^  (95% CI) | - | 0.08  (-0.03, 0.19) | 0.00  (-0.06, 0.07) | 0.02  (0.02, 0.03) | 0.00  (-0.02, 0.02) | 0.01  (-0.01, 0.02) |
|  |  |  |  |  |  |  |
| **Allergic rhinitis and type 1 diabetes** |  |  |  |  |  |  |
| Number of concordant pairs | - | 27 | 70 | 6,782 | 1,295 | 1,381 |
| Concordance rate**^*^**  (95% CI) | - | 0.01  (0.00, 0.01) | 0.01  (0.00, 0.01) | 0.01  (0.01, 0.01) | 0.01  (0.01, 0.01) | 0.01  (0.01, 0.01) |
| Phenotypic correlation**^†^**  (95% CI) | 0.06  (0.05, 0.06) | 0.05  (-0.05, 0.15) | 0.04  (-0.01, 0.10) | 0.05  (0.05, 0.06) | 0.06  (0.04, 0.07) | 0.06  (0.04, 0.07) |
| Cross-relative cross-trait correlation^‡^  (95% CI) | - | 0.07  (-0.03, 0.17) | 0.00  (-0.06, 0.06) | 0.02  (0.02, 0.03) | 0.01  (0.00, 0.03) | 0.00  (-0.01, 0.01) |
|  |  |  |  |  |  |  |
| **Eczema and type 1 diabetes** |  |  |  |  |  |  |
| Number of concordant pairs | - | 6 | 42 | 3,597 | 654 | 756 |
| Concordance rate**^*^**  (95% CI) | - | 0.00  (0.00, 0.01) | 0.01  (0.00, 0.01) | 0.01  (0.01, 0.01) | 0.01  (0.01, 0.01) | 0.01  (0.01, 0.01) |
| Phenotypic correlation**^†^**  (95% CI) | 0.05  (0.04, 0.06) | -0.01  (-0.13, 0.11) | 0.10  (0.04, 0.16) | 0.05 (0.04, 0.06) | 0.05  (0.04, 0.07) | 0.04  (0.02, 0.06) |
| Cross-relative cross-trait correlation^‡^  (95% CI) | - | -0.11  (-0.24, 0.02) | 0.02  (-0.04, 0.09) | 0.00  (-0.01, 0.01) | 0.01  (-0.01, 0.03) | 0.01  (-0.01, 0.03) |
|  |  |  |  |  |  |  |

^*^The concordance rate (bivariate) is the proportion of individuals with one disease if the relative had the other disease

^†^The phenotypic correlation is the correlation between the two diseases, within the same individual

^‡^The cross-relative cross-trait correlation is the correlation between the two diseases, between individuals

# D. References

1. Lloyd-Jones LR, Zeng J, Sidorenko J, Yengo L, Moser G, Kemper KE, et al. Improved polygenic prediction by Bayesian multiple regression on summary statistics. Nature Communications. 2019;10(1):1–11.

2. Ludvigsson JF, Almqvist C, Bonamy AKKE, Ljung R, Michaëlsson K, Neovius M, et al. Registers of the Swedish total population and their use in medical research. Eur J Epidemiol. 2016;31(2):125–36.

3. Ekbom A. The Swedish Multi-generation Register. Methods Mol Biol. 2011;675:215–20.

4. Ludvigsson JF, Andersson E, Ekbom A, Feychting M, Kim JL, Reuterwall C, et al. External review and validation of the Swedish national inpatient register. BMC Public Health. 2011;11(1):450.

5. Wettermark B, Hammar N, Fored CM, Leimanis A, Olausson PO, Bergman U, et al. The new Swedish Prescribed Drug Register Opportunities for pharmacoepidemiological research and experience from the first six months. Pharmacoepidemiol Drug Saf. 2007;16(7):726–35.

6. Zagai U, Lichtenstein P, Pedersen NL, Magnusson PKE. The Swedish Twin Registry: Content and Management as a Research Infrastructure. Twin Res Hum Genet. 2019;22(6):672–80.

7. Magnusson PKE, Almqvist C, Rahman I, Ganna A, Viktorin A, Walum H, et al. The Swedish Twin Registry: Establishment of a Biobank and Other Recent Developments. Twin Res Hum Genet. 2013;16(1):317–29.

8. Örtqvist AK, Lundholm C, Wettermark B, Ludvigsson JF, Ye W, Almqvist C. Validation of asthma and eczema in population-based Swedish drug and patient registers. Pharmacoepidemiol Drug Saf. 2013;22(8):850–60.

9. Henriksen L, Simonsen J, Haerskjold A, Linder M, Kieler H, Thomsen SF, et al. Incidence rates of atopic dermatitis, asthma, and allergic rhinoconjunctivitis in Danish and Swedish children. Journal of Allergy and Clinical Immunology. 2015;136(2):360-366.e2.

10. Rawshani A, Landin-Olsson M, Svensson AM, Nyström L, Arnqvist HJ, Bolinder J, et al. The incidence of diabetes among 0–34 year olds in Sweden: new data and better methods. Diabetologia. 2014;57(7):1375–81.

11. Ferreira MAR, Mathur R, Vonk JM, Szwajda A, Brumpton B, Granell R, et al. Genetic Architectures of Childhood- and Adult-Onset Asthma Are Partly Distinct. The American Journal of Human Genetics. 2019;104(4):665–84.

12. Zhou W, Kanai M, Wu KHH, Rasheed H, Tsuo K, Hirbo JB, et al. Global Biobank Meta-analysis Initiative: Powering genetic discovery across human disease. Cell Genomics. 2022;2(10):100192.

13. Waage J, Standl M, Curtin JA, Jessen LE, Thorsen J, Tian C, et al. Genome-wide association and HLA fine-mapping studies identify risk loci and genetic pathways underlying allergic rhinitis. Nature Genetics 2018 50:8. 2018;50(8):1072–80.

14. Sliz E, Huilaja L, Pasanen A, Laisk T, Reimann E, Mägi R, et al. Uniting biobank resources reveals novel genetic pathways modulating susceptibility for atopic dermatitis. Journal of Allergy and Clinical Immunology. 2022;149(3):1105-1112.e9.

15. Chiou J, Geusz RJ, Okino ML, Han JY, Miller M, Melton R, et al. Interpreting type 1 diabetes risk with genetics and single-cell epigenomics. Nature. 2021;594(7863):398–402.
